# Public Attitudes toward Pharmacogenomic Testing and Establishing a Statewide Pharmacogenomics Database in the State of Minnesota

**DOI:** 10.3390/jpm12101615

**Published:** 2022-09-30

**Authors:** Lusi Zhang, Pamala A. Jacobson, Alyssa N. K. Johnson, David B. Gregornik, Steven G. Johnson, Catherine A. McCarty, Jeffrey R. Bishop

**Affiliations:** 1 Department of Experimental and Clinical Pharmacology, College of Pharmacy, University of Minnesota, Minneapolis, MN 55455, USA; 2 Department of Medical Genetics and Genomics, Children’s Minnesota, Minneapolis, MN 55404, USA; 3 Institute for Health Informatics, University of Minnesota, Minneapolis, MN 55455, USA; 4 Department of Family Medicine and Biobehavioral Health, Medical School, University of Minnesota, Duluth, MN 55812, USA; 5 Department of Psychiatry and Behavioral Sciences, University of Minnesota Medical School, Minneapolis, MN 55455, USA

**Keywords:** public attitudes, pharmacogenomics, pharmacogenomic testing, Minnesota, implementation

## Abstract

The clinical adoption and implementation of pharmacogenomics (PGx) beyond academic medical centers remains slow, restricting the general population from benefitting from this important component of personalized medicine. As an initial step in the statewide initiative of PGx implementation in Minnesota, we engaged community members and assessed attitudes towards PGx testing and acceptability of establishing a secure statewide PGx database for clinical and research use among Minnesota residents. Data was collected from 808 adult attendees at the 2021 Minnesota State Fair through an electronic survey. Eighty-four percent of respondents felt comfortable getting a PGx test for clinical care. Most respondents trusted health professionals (78.2%) and researchers (73.0%) to keep their PGx data private. The majority expressed their support and interest in participating in a statewide PGx database for clinical and research use (64–72%). Higher acceptability of the statewide PGx database was associated with younger age, higher education, higher health literacy, having health insurance, and prior genetic testing. The study sample representing Minnesota residents expressed high acceptability of receiving PGx testing and willingness to participate in PGx data sharing for clinical and research use. Community support and engagement are needed to advance PGx implementation and research on the state scale.

## 1. Introduction

Pharmacogenomics (PGx) is a rapidly growing approach to improving treatment precision using genetic factors to guide drug selection or dosing [1,2]. It has great potential to facilitate personalized pharmacotherapy, reduce adverse reactions, and maximize effectiveness based on a person’s genetic profile [3]. Nearly 30 years of research supports the transition of PGx research into clinical application. As a result, over 40 commercial laboratories currently offer PGx tests in the United States, with many guiding the use of medications in mental health, oncology, cardiology, infectious disease, neurology, and other therapeutic areas [4]. The growing utilization of commercially available PGx products is also evident in many other countries across the globe. Accumulating evidence-based and peer-reviewed PGx clinical guidelines and supporting resources have been developed by the Clinical Pharmacogenetics Implementation Consortium (CPIC), the Dutch Pharmacogenetics Working Group (DPWG), American College of Medical Genetics and Genomics (ACMG), Association of Molecular Pathology (AMP), American College of Pathologists (CAP), Ubiquitous Pharmacogenomics (U-PGx), and other professional societies [5,6,7,8,9,10]. Since 2009, CPIC has published 26 guidelines that cover 93 drugs and 25 genes across therapeutic specialties, which continue to be regularly updated [11]. In the U.S., CPIC guidelines along with the U.S. Food and Drug Administration (FDA) product labeling [12] support PGx implementation in routine clinical care.

Progress in PGx implementation in the U.S. has been made within a few research hospitals and health systems [13,14,15,16]. However, the adoption and implementation of PGx into clinical workflows beyond specialized centers and programs have been slow, which limits the ability of the general population to benefit from this important component of precision medicine [17]. Barriers that delay the process include, but are not limited to, a lack of education and awareness, infrastructure needs, costs, perceptions about PGx testing, and data storage [18,19]. The barriers to implementing PGx in the community can be overcome but require the engagement of multiple stakeholders and a collaborative initiative to share knowledge and practices [20].

With the overarching goal of having the broader community benefit from the PGx-guided individualized medication management, the University of Minnesota (UMN), in conjunction with statewide partners, has established an initiative to conceptualize and implement a framework for PGx implementation at a statewide scale in Minnesota [21]. By leveraging resources across clinical and research institutions, community pharmacies, and commercial labs, the ongoing UMN-supported “Grand Challenge-Implementing Pharmacogenomics at a Statewide Scale” project represents a one-of-a-kind implementation landscape supporting the clinical adoption of PGx across the entire state and engaging stakeholders and policymakers to facilitate this statewide approach to implementation [21,22]. The development of multi-institution clinical decision support systems to deliver PGx test results and interpretation [23], the initiation of a PGx Project ECHO (Extension for Community Healthcare Outcomes) program [24], and the Minnesota Department of Health supported PGx Certificate program for pharmacists to support workforce education and PGx implementation in rural and underserved communities [25] are examples of ongoing endeavors within this initiative.

One mission of the MN statewide PGx implementation is to develop the health information technology architecture required to leverage PGx for optimal medication use. The framework of a secure, centralized, patient-centered database to store PGx information has been conceptualized [23], which will allow patients and consumers to control and share their PGx information with healthcare professionals and advance future discovery in PGx research. As an initial step, we engaged community members to identify opportunities and barriers. To our knowledge, this represents a unique effort to systematically assess and engage public stakeholders. The present study was designed to determine public attitudes toward PGx testing, the acceptability of establishing a PGx database for clinical and research use, and the willingness to participate among Minnesota residents.

## 2. Materials and Methods

### 2.1. Eligibility and Recruitment

The study team conducted a cross-sectional survey at the Driven to Discover (D2D) Research Facility at the Minnesota State Fair over the course of five days between 26 August and 5 September 2021. The Minnesota State Fair has the highest daily state fair attendance in the United States with over 1.3 million attendees in 2021 [26]. The D2D program was launched in 2014 by the University of Minnesota to connect researchers with the broader Minnesota community [27].

Potential participants who self-identified as Minnesota residents 18 years of age or older were invited to participate in the “How Do Your Genes Fit” study by the research team. Research team members introduced the study, screened for eligibility, reviewed the consent form with participants, and answered questions to clarify survey procedures before participation. Exclusion criteria included a lack of English proficiency and not being able to provide informed consent. Eligible participants who decided to consent were instructed to take an anonymous survey on Apple iPads, which took approximately 15 min to complete. All participants received a University of Minnesota backpack (valued at $2), pill organizer, or other items of equal value as a thank you for their time upon completion of the survey. Participant responses were captured using Research Electronic Data Capture (REDCap), which uses a database and secure web interface to store participant responses. This study was reviewed and approved by the University of Minnesota Institutional Review Board #00009777 on 7/20/21.

### 2.2. Survey Design and Measures

The survey consisted of 44 to 53 questions, depending on the skip pattern, related to participant sociodemographic and health-related characteristics, health literacy, and personal beliefs regarding PGx testing and research. An additional set of 15 items included questions used to assess PGx literacy, which was used to validate the Minnesota Assessment of Pharmacogenomic Literacy (MAPL) [28].

Sociodemographic variables included age, gender, race, ethnicity, educational attainment, and geographic region of residence (i.e., metro vs. non-metro). A multiple-choice question with different age ranges was added to collect age information after the first day of survey administration due to the trend of a large proportion of missing age values in the free text field. The free-text age responses were characterized into age ranges when applicable during data cleaning. Respondents reported ZIP codes that were used by the study team to determine geographical locations and metro/non-metro classification according to the 2010 Rural-Urban Commuting Area (RUCA codes) [29]. Health-related characteristics included insurance coverage, whether they had a primary care provider, recent medication experience, and whether they had previously had commercial genetic or PGx testing. Health literacy was assessed with questions from the All Aspects of Health Literacy Scale (AAHLS) [30]. The AAHLS total score was calculated by summing the score across 10 questions of functional, communicative, and critical health literacy, which were rated on a three-point Likert scale (0, 1, 2). The AAHLS total score ranges from 0 to 20, with a higher score indicating higher health literacy (see Allen et al., 2022 [28] for details on AAHLS scoring).

To assess public acceptability of PGx testing, eight questions were asked on a five-point Likert scale ranging from “Strongly disagree” to “Strongly agree” assessing respondents’ comfort level of receiving a PGx test (two questions), level of trust to maintain PGx data privacy (five questions), and perception of data sharing in PGx research (two questions). Four questions were asked on a three-point scale (Yes/No/I don’t know) to measure the acceptability of establishing a secure statewide PGx database for clinical and research purposes and willingness to participate with detailed explanations of the purpose of this PGx data shelter, clinical use, research use, and activities involved. To assess willingness to pay for PGx testing, respondents were asked to choose “the amount I would pay out-of-pocket for pharmacogenomic test” from seven prespecified options: “I would not pay any”, “$1–$49”, “$50–$149”, “$150–$249”, “$250–$499”, “$500–$999”, and “$1000+”. The complete list of PGx-related survey questions is summarized in Supplementary Materials Supplement I.

### 2.3. Data Analysis

**Figure 1:**
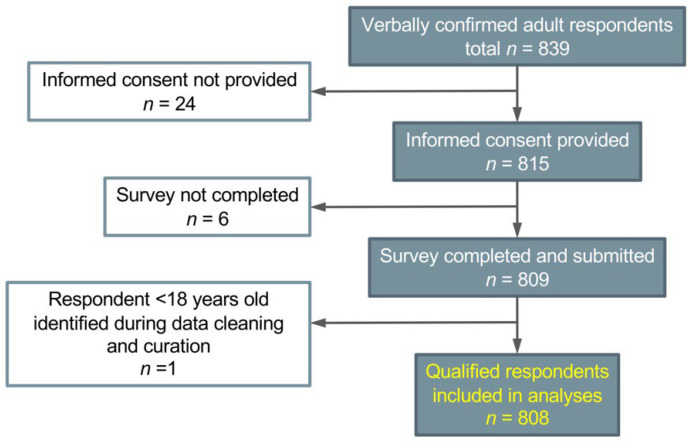
Flow diagram of study participants.

Data cleaning and analyses were performed in R version 4.0.2. The data cleaning procedure is illustrated in the study flowchart (Figure 1). Qualified respondents’ characteristics were summarized with descriptive statistics using frequencies and means. Medication safety and efficacy concerns were examined in relation to the total number of prescription medications with the Chi-square test. Relationships between whether participants would value and/or be willing to participate in a statewide database for clinical and research purposes and sociodemographic and health-related characteristics were examined by pairwise Spearman correlation analyses followed by multivariable logistic regression analyses. Health literacy (AAHLS total score) was compared with the acceptability of the statewide PGx database using the ANOVA test and *t*-tests for post-hoc pairwise comparisons.

## 3. Results

### 3.1. Participant Characteristics

Among the 808 eligible respondents included in the analyses, 63.2% self-identified as women (Table 1). The median age range was 42–53 years. The majority of respondents (84.3%) self-identified as White and non-Hispanic/Latino (92.6%). Over 90% of respondents had attained some level of college education and 22% completed postgraduate education. Over 94% of respondents were covered by at least one type of health insurance plan and nearly 84% reported having a primary care provider. Of note, nearly 18% of respondents had previously received commercial genetic testing.

### 3.2. Participant Medication Experience

**Figure 2:**
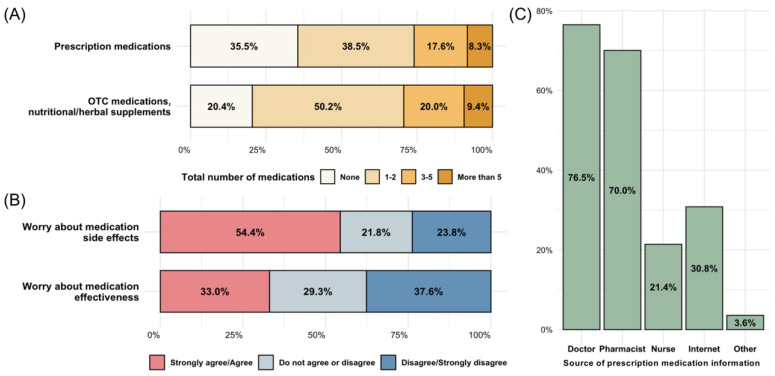
Medication experience among survey respondents. (**A**) Prescription and non-prescription medication use in the last 30 days; (**B**) Concerns about medication safety and effectiveness; (**C**) Sources to receive information about prescription medications. OTC: over-the-counter.

The total numbers of prescription and non-prescription medications that participants reported taking in the past 30 days are summarized in Figure 2A. The majority of the respondents reported taking at least one prescription (64.5%) or non-prescription medication (79.6%). Approximately 26% reported taking three or more prescription medications over the past month. When asked about their concerns regarding medications, 54.4% and 33.0% acknowledged their worry about medications’ side effects and effectiveness, respectively (Figure 2B). Respondents who took three or more prescription medications were more likely to worry about medication side effects (X^2^ = 21.969, df = 4, *p* < 0.001) and effectiveness (X^2^ = 18.112, df = 4, *p* = 0.001) (Supplementary Figure S1). Most respondents received information about prescriptions from physicians (76.5%) and pharmacists (70.0%) and to a lesser extent from nurses (21.4%) and the internet (30.8%) (Figure 2C).

### 3.3. Acceptability and Perception of PGx Testing, Data Privacy, and Research

**Figure 3:**
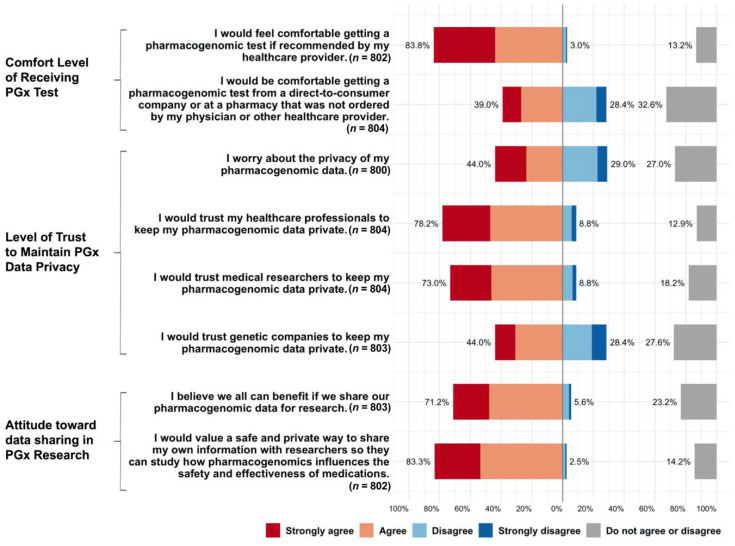
Acceptability of receiving pharmacogenomic (PGx) tests and attitudes toward data privacy and research among survey respondents.

The total response rates for all questions about attitudes toward PGx were above 99%. Most respondents felt comfortable obtaining a PGx test if recommended by their healthcare provider (83.8%). In contrast, less than half of respondents (39.0%) felt comfortable obtaining a PGx test from a direct-to-consumer company or at a pharmacy that was not ordered by their healthcare provider. When asked about PGx data privacy, 44.0% of respondents acknowledged their worry about the privacy of their data, but most responded that they would trust healthcare professionals (78.2%) and researchers (73.0%) to keep their PGx data private. In total, 44% trusted genetic companies to keep PGx data private and 28.4% did not. The study cohort expressed high acceptability of PGx data sharing for research, with 71.2% of respondents believing that we can all benefit from it. In particular, 83.3% of respondents valued a safe and private way to share their own information with researchers to study how PGx influences medication safety and effectiveness (Figure 3).

**Figure 4:**
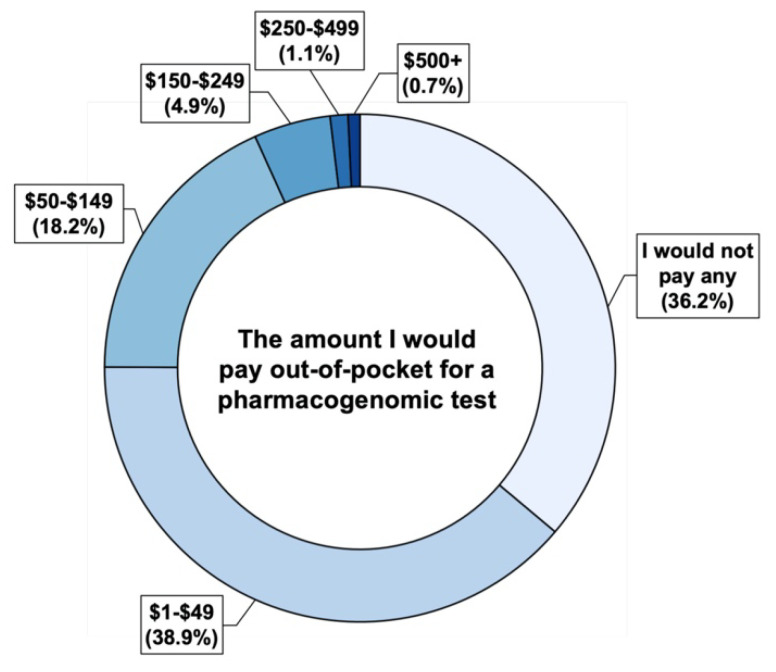
The expected out-of-pocket spending on pharmacogenomic (PGx) testing among survey respondents.

The expected out-of-pocket spending on PGx testing among respondents is summarized in Figure 4. Over 36% of respondents were not willing to incur any out-of-pocket cost for PGx testing. Among all participants, 38.9%, 57.1%, and 62% were willing to pay less than $50, less than $150, and less than $250 for PGx testing, respectively.

### 3.4. Attitudes toward Developing a Statewide PGx Database

**Figure 5:**
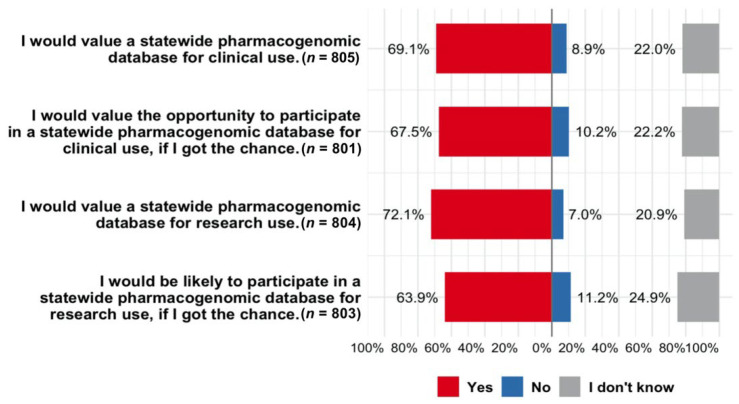
Acceptability of a statewide pharmacogenomic shelter for clinical and research purposes. Complete survey questions are: (1) I would value a statewide pharmacogenomic database for clinical use. This database would allow pharmacogenomic test results to be available to doctors and pharmacists, with an individual’s permission, so they can check genes against medications prescribed to their patients for safety and effectiveness. (2) I would value the opportunity to participate in a statewide pharmacogenomic database for clinical use, if I got the chance. “Clinical use” means my information could be provided to doctors and pharmacists, with my permission, so they could check my genes against drugs prescribed to me for safety and effectiveness. (3) I would value a statewide pharmacogenomic database for research use. “Research use” means that individuals could allow their information to be provided to researchers, so they can study how pharmacogenomics influences safety and effectiveness of medications. (4) I would be likely to participate in a statewide pharmacogenomic database for research use, if I got the chance. This would allow my information to be provided to researchers, with my permission, so they can study how pharmacogenomics influences the safety and effectiveness of medications.

The majority of respondents expressed positive attitudes toward establishing a statewide PGx database for clinical use and research (Figure 5). Specifically, 69.1% and 72.1% of respondents believed that they would value a statewide PGx database for clinical and research use, respectively. In terms of the willingness to participate, 67.5% and 63.9% of respondents stated that they would be likely to participate in a statewide PGx database for clinical and research use, respectively. When examining how sociodemographic and health-related characteristics were related to attitudes toward a state-wide PGx database, exploratory pairwise analyses identified significant correlations with education, having health insurance, and having prior genetic testing performed (Table 2). Willingness to participate was negatively correlated with age. Support for a statewide PGx database for clinical use was negatively correlated with non-White ancestry and non-metro primary residence. Support for a database for research was negatively correlated with a non-metro primary residence. When examining the characteristics together using multivariable regression, higher education level was significantly associated with being supportive of the establishment of a statewide PGx database for both clinical and research use and willingness to participate (Table 3). Respondents who had commercial genetic testing performed in the past were more likely to support and be willing to participate in the statewide PGx database. Younger individuals (18–29 years of age) were more willing to participate than those 30 years or older. Having health insurance was also associated with positive attitudes toward the statewide PGx database. The AAHLS total scores were higher among respondents who were supportive of and willing to participate in the statewide PGx database than those who did not, indicating a relationship between higher health literacy and acceptability of the statewide PGx database for clinical and research use (Supplemental Figure S2).

## 4. Discussion

In the present study, we investigated the attitudes of community stakeholders toward PGx testing and a statewide PGx database Minnesota. This sample from a large community-based population outside of the healthcare setting expressed a high level of comfort in receiving PGx testing, and willingness to participate in PGx research. Participants largely responded that they would value the development of a statewide PGx database for research and to support the portability of PGx results for clinical care so doctors and pharmacists could check their genes against prescribed medications for safety and effectiveness. We also identified key sociodemographic determinants of the level of support and willingness, including age, education attainment, health literacy, insurance coverage, and prior genetic testing. These findings demonstrate the public’s support and acceptability for a statewide PGx initiative to advance cutting-edge clinical care and research. Our approach to engaging community member stakeholders may serve as a model for other states interested in pursuing statewide PGx initiatives.

### 4.1. Acceptability of Receiving PGx Testing for Clinical Use

The acceptability of obtaining PGx testing among Minnesota residents is consistent with the overall positive attitude toward PGx in prior studies in the U.S. and other countries [31,32,33,34,35]. For example, a recent survey conducted in Spain found that over 68% of Spanish patients support the universal population PGx testing, with age and education as predictors of the level of acceptability [35]. In a previous study performed with a sample of the US public, 70 to 92% of respondents (total *n* = 1139) expressed interest in pursuing PGx testing to predict medication effectiveness and serious side effects or assist in dosing selection in general [36]. They identified an association between having medication side effects in the past and the acceptability of receiving PGx testing, which was also confirmed by other studies. Among Minnesota respondents in our study, over half acknowledged their worry about medication side effects or effectiveness, especially those who took three or more prescription medications. In contrast to some previous reports, these factors were not primary determinants of the acceptability of PGx in this study sample.

We observed differential patterns of acceptability for PGx testing based on how testing services would be obtained. The level of comfort for obtaining PGx testing recommended by healthcare providers was over two times higher than that if the test was obtained from a direct-to-consumer company or at a pharmacy. Among multiple barriers to PGx implementation, concerns about data privacy and confidentiality notably dampen customers’ enthusiasm to pursue PGx testing [33]. In our study, nearly half of the respondents expressed general worry about the privacy of their PGx data, but importantly, they also reported a high level of trust in healthcare professionals’ ability to keep their PGx data private. These findings highlight the importance of trust and rapport in the relationship between patients and clinicians when delivering PGx testing services. Consistent with our findings, convergent evidence from previous studies confirmed customers’ strong desire to receive PGx testing from healthcare professionals who were capable of explaining the test results and interpreting the implications for medication selections through effective communication and education [32,33,37]. This expectation of community stakeholders underscores the importance of ensuring that PGx education for healthcare professionals and the integration of PGx education programs are incorporated into clinical implementation programs.

### 4.2. Financial Perspectives on PGx Testing

Early implementation efforts of PGx testing largely relied on institutional support and external funding mechanisms to cover the costs due to the limited insurance coverage and reimbursement of PGx testing [18,38,39]. The cost of testing has been repeatedly identified as a major barrier for expansion and large-scale clinical adoption [13,40,41,42]. Therefore, having a better understanding of the public’s expectations and perspectives on the cost of PGx testing and the willingness-to-pay is an essential step in addressing the financial infrastructure of widespread PGx adoption.

The majority of respondents in our study expected no or low (less than $50) out-of-pocket spending on PGx testing. In a survey conducted among 869 patients from the Mayo Clinic Right Drug, Right Dose, Right Time (RIGHT) Protocol study in 2014, 42% of the respondents were unwilling to incur any out-of-pocket costs for PGx testing [13]. This proportion of the RIGHT patient population was higher than what was observed in our community-based population (36%) but comparable in general. Financial barriers were previously identified as a key indicator of unwillingness to pay for PGx testing [18]. Patients and consumers are more likely to pursue PGx testing if the cost is covered by insurance [18,43]. This is a particularly important consideration in Minnesota based on the limited regional coverage of PGx testing [21]. In the face of the shortage of insurance coverage for PGx testing, these findings collectively support the need for large-scale programs, such as a statewide initiative, to address the infrastructure disparity, allocate resources to work on this problem, and change policies to make PGx testing more accessible to Minnesotans.

### 4.3. Attitudes toward PGx Data Sharing for Research

A positive attitude toward data sharing in PGx research was evident among our study participants. Over 70% of participants trusted PGx data privacy in research and believed that the community would benefit from sharing PGx data for research purposes. It is also notable that over 83% of individuals were willing to contribute their personal data in a safe and private way to help advance research examining PGx’s impact on the safety and effectiveness of medications. This highlights the importance of raising awareness of the value that research can bring to the community by engaging local residents in PGx research. The risk of data privacy and misuse of personal information was seen as a major concern of potential research participants that may outweigh their perception of the importance of contributing to biomedical research and adversely impact their decision to participate [44]. With effective data protection and thoughtful strategies for ensuring safe and private data collection, community members are likely to be more willing to participate in PGx research that is beneficial for the community.

### 4.4. Community Support for Establishing a Statewide PGx Database

A positive attitude toward data sharing in PGx research was evident among our study participants. The majority of respondents (around 70%) were in support of this initiative and stated that they would value the opportunity to share their PGx information with clinicians to support clinical practice and advance future research. The acceptability of this statewide PGx database is comparable with public attitudes toward nationwide precision/personalized medicine research or biobanks assessed by prior studies [45,46]. Interestingly, the willingness to participate in this statewide PGx initiative was greater than that of the willingness to participate in precision medicine research assessed among a similar population in 2017 [47]. Perhaps some broader components of precision medicine, such as medical genetics, are associated with uncertainties as compared to PGx, which focuses on medications with obvious benefits to the individual. Of note, 20% of respondents in our study could not decide on whether they wanted to participate in the PGx database or not, which is over twice as high as the proportion of individuals who were unwilling to participate. The neutral attitude could be an indicator of uncertainty or a lack of clarity about the value of the statewide PGx initiative or other concerns not assessed in our survey that outweigh the intention to participate. Future efforts are needed to raise the awareness and understanding of PGx with targeted education to enhance participant and public involvement in the large-scale PGx initiative.

When examining the potential influences of sociodemographic factors, younger age, higher education attainment, and having health insurance were found to be the most associated with a higher willingness to participate in a statewide PGx database. Individuals who self-identified as White expressed more support for the clinical use of a PGx database. These factors were also reported by previous studies to influence attitudes toward or willingness to pursue PGx [4,35] and participate in research or biobank initiatives of PGx and precision medicine [45,46,47,48]. Individuals with genetic testing performed in the past would be more likely to participate in the statewide PGx initiative, likely motivated by a standing interest in genetic testing and acquired knowledge from previous experiences. In addition, higher health literacy, as assessed by the AAHLS, was also associated with greater willingness to participate in the statewide PGx initiative. Assessing clients’ health literacy has been recognized as an important component of genetic counseling for disease risks [49]. In PGx, a handful of studies suggest that individuals with higher health literacy are more likely to have a better understanding of the test results [50]. Yet, the role of health literacy in clinical PGx service and research has only been sparsely explored. The Minnesota Assessment of Pharmacogenomic Literacy (MAPL) was recently developed and validated by our team, aiming to quantify individuals’ PGx literacy to address the needs of both clinical and research settings [28]. Additional efforts are needed to examine and improve health literacy in both the clinical and research contexts of PGx in the near future. For example, the PGx data repository could be linked to existing data shelters to answer questions about clinical outcomes and gain further insights into who may gain the most benefits from PGx testing.

### 4.5. Study Limitations and Recommendations for Future Research

Several limitations are important to consider when interpreting and generalizing these findings. The convenience sampling used in this study limits the generalizability of the study sample. The sample was recruited from a large public venue in the Minneapolis/St. Paul metropolitan area, resulting in a predominantly insured population with high education levels and good healthcare access. A relatively smaller proportion of participants were from non-metro and rural areas. The education attainment level of the study sample was higher than broader statewide statistics [51]. Specific racial groups, such as persons of African ancestry, were underrepresented in the sample with reference to the state demographics for Minnesota [52]. Ascertainment bias may also have occurred since those individuals who had an interest in medical research, especially those with higher education attainment, may have been more likely to participate in this study. Future studies are needed to further assess public perspectives in non-metro areas and to increase recruitment of underserved and minority populations to characterize their attitudes toward PGx and the statewide PGx initiative. This is achievable through ongoing stakeholder outreach through the University of Minnesota’s urban and rural health programs and an established infrastructure for community-based participatory research for PGx and precision medicine [53,54].

## 5. Conclusions and Implications

This study sample representing Minnesota residents expressed a high level of acceptability to receiving PGx testing, sharing PGx data for clinical purposes, and participation in PGx research. The population also showed enthusiasm and support for the establishment of a statewide PGx initiative for clinical and research purposes. Community support and engagement are crucial for advancing PGx implementation and research at the state scale. Approaching PGx at a statewide scale will eventually advance the health of all Minnesota residents, promote health equity, reduce health disparities, and support our Minnesota institutions in research and healthcare innovation.

## Figures and Tables

**Table 1:** Characteristics of survey respondents (*n* = 808) ^a^.

	*n*	%
Gender		
Men	290	35.9
Women	510	63.2
Non-binary	3	0.4
Unknown/Prefer not to answer	4	0.5
Age		
18–29	203	27.5
30–41	103	14.0
42–53	147	19.9
54–65	195	26.4
66–77	82	11.1
78+	8	1.1
Race		
American Indian or Alaska Native	6	0.7
Asian	59	7.3
Black or African American	8	1.0
Native Hawaiian or Pacific Islanders	2	0.2
White	679	84.3
Multiracial	18	2.2
Other	15	1.9
Unknown/Prefer not to answer	18	2.2
Hispanic, Latino, or Spanish origin		
Yes	44	5.5
No	746	92.6
Unknown/Prefer not to answer	16	2.0
Education attainment		
No college	78	9.7
Some college, no degree	160	19.8
Associate degree	107	13.2
Bachelor’s degree	286	35.4
Master’s degree	117	14.5
Doctoral degree	58	7.2
Unknown/Prefer not to answer	2	0.2
Geographical regions ^b^		
Metro	713	90.5
Non-metro	75	9.5
Having health insurance		
No	46	5.7
Yes	757	94.3
Insurance type		
Public health plan (Medicare/Medicaid)	183	
Private health plan	132	
Employer health plan	546	
Military health plan	22	
Indian Health Service health plan	1	
Having primary care provider		
Yes	672	83.7
No	131	16.3
Having commercial genetic testing performed in the past		
Yes	141	17.5
No	663	82.5

^a^ The percentage of missing values for all key sociodemographic variables were less than 2%, except for age (8.7% missing response rate). ^b^ Metro vs. non-metro classification was characterized using the ZIP codes collected from respondents and according to the 2010 Rural-Urban Commuting Area Codes (https://www.ers.usda.gov/data-products/rural-urban-commuting-area-codes/ accessed on 18 July 2022).

**Table 2:** Pairwise Spearman correlations of the acceptability of a statewide pharmacogenomic database and sociodemographic and health-related characteristics.

	Support Statewide PGx Database for Clinical Use			Willingness to Participate in Statewide PGx Database for Clinical Use			Support Statewide PGx Database for Research Use			Willingness to Participate in Statewide PGx Database for Research Use		
	r ^a^	*p* Value	*n*	r	*p* Value	*n*	r	*p* Value	*n*	r	*p* Value	*n*
Gender	−2.36 × 10^−4^	0.995	804	−2.90 × 10^−4^	0.993	800	3.85 × 10^−3^	0.913	803	0.047	0.180	802
Age	0.018	0.633	736	−0.087	0.018	732	−0.059	0.110	735	−0.073	0.049	734
White vs. non-White	−0.104	0.003	802	−0.089	0.012	798	−0.030	0.396	801	−0.032	0.366	800
Metro vs. non-metro	−0.071	0.047	785	−0.053	0.136	782	−0.096	0.007	784	−0.052	0.144	783
Education	0.175	5.99 × 10^−7^	805	0.150	1.96 × 10^−5^	801	0.187	9.13 × 10^−8^	804	0.155	1.04 × 10^−5^	803
Having health insurance	0.148	2.67 × 10^−5^	800	0.174	8.35 × 10^−7^	796	0.147	3.20 × 10^−5^	799	0.128	2.78 × 10^−4^	798
Having primary care provider	0.054	0.127	800	0.011	0.753	796	0.035	0.327	799	0.040	0.255	798
Having prior genetic testing performed	0.090	0.011	803	0.103	3.51 × 10^−3^	799	0.074	0.036	802	0.108	2.14 × 10^−3^	801
Total number of prescription medications	0.039	0.269	802	−0.009	0.806	798	0.013	0.722	801	0.022	0.540	800

^a^. r indicates the Spearman’s rank coefficient of correlation; *n* indicates the size of the pairwise complete observations.

**Table 3:** Multivariable logistic regression models of the acceptability of a statewide pharmacogenomic database on sociodemographic and health-related characteristics.

Outcome Variable ^a^	Model 1 ^b^ Support a Statewide PGx Database for Clinical Use		Model 2 Would Participate in Statewide PGx Database for Clinical Use		Model 3 Support a Statewide PGx Database for Research Use		Model 4 Would Participate in Statewide PGx Database for Research Use	
	Coefficient	SE	Coefficient	SE	Coefficient	SE	Coefficient	SE
Female (reference: Male)	−0.236	0.189	−0.125	0.186	−0.094	0.193	0.128	0.178
Age (reference: 18–29 years)								
30–41	−0.190	0.289	−0.685 *	0.293	−0.335	0.309	−0.450	0.283
42–53	−0.384	0.271	−0.794 **	0.279	−0.665 *	0.286	−0.571 *	0.265
54–65	−0.067	0.255	−0.639 *	0.260	−0.510	0.264	−0.405	0.246
66–77	−0.243	0.333	−1.091 **	0.329	−0.522	0.345	−0.888 **	0.314
78+	−0.394	0.945	0.446	1.180	−1.464	0.882	0.810	1.148
Non-White (reference: White)	−0.597 *	0.239	−0.501 *	0.244	−0.179	0.258	−0.030	0.243
Non-metro (reference: Metro)	−0.488	0.292	−0.088	0.299	−0.458	0.296	−0.154	0.293
Education (reference: No college)								
Some college, no degree	1.018 **	0.336	1.087 **	0.341	0.738 *	0.331	0.915 **	0.339
Associate degree	1.250 **	0.365	1.045 **	0.365	0.921 *	0.360	0.992 **	0.362
Bachelor’s degree	1.613 ***	0.322	1.610 ***	0.326	1.487 ***	0.321	1.509 ***	0.323
Master’s degree	1.454 ***	0.376	1.433 ***	0.375	1.376 ***	0.380	1.151 **	0.367
Doctoral degree	1.935 ***	0.479	1.715 ***	0.455	2.136 ***	0.527	2.495 ***	0.511
Having health insurance	0.778	0.406	1.344 **	0.416	0.870 *	0.404	1.019 *	0.413
Having primary care provider	0.196	0.255	−0.053	0.265	0.245	0.265	0.122	0.248
Having commercial genetic testing performed in the past	0.484	0.250	0.757 **	0.253	0.472	0.258	0.695 **	0.239
Total number of prescription medications (reference: None)								
1–2	0.338	0.210	0.183	0.211	0.247	0.219	0.283	0.201
3+	0.031	0.235	−0.146	0.235	−0.043	0.242	0.077	0.228

^a^. Complete survey questions for each variable: Model 1: “I would value a statewide pharmacogenomic database for clinical use.” Model 2: “I would value the opportunity to participate in a statewide pharmacogenomic database for clinical use, if I got the chance.” Model 3: “I would value a statewide pharmacogenomic database for research use.” Model 4: “I would be likely to participate in a statewide pharmacogenomic database for research use, if I got the chance.” Responses for each question were dichotomized as “Yes” vs. “No/I don’t know”. Statistical significance: * *p* < 0.05, ** *p* < 0.01, *** *p* < 0.001. PGx: Pharmacogenomic(s). ^b^. A total of four separate multivariable logistic regression models were built, with each including an outcome measuring the acceptability of a statewide pharmacogenomic database and the selected characteristics as predictors.

## Data Availability

The data presented in this study are available on request from the corresponding author. The data are not publicly available in accordance with the approval of the Institutional Review Board.

## Supplementary Materials

The following supporting information can be downloaded at: https://www.mdpi.com/article/10.3390/jpm12101615/s1, Supplement I: Survey questions regarding the acceptability of receiving pharmacogenomic (PGx) tests, attitudes toward data privacy and research, and acceptability of a statewide PGx database for clinical and research purposes; Figure S1: Safety and effectiveness concerns about medication use by the total number of prescription medications; Figure S2: The associations between health literacy, assessed by AAHLS total score, and acceptability of a statewide pharmacogenomic database for clinical and research purposes among survey respondents (*n* = 763).
