# Supplementary material for: Public Attitudes toward Pharmacogenomic Testing and Establishing a Statewide Pharmacogenomics Database in the State of Minnesota
Source: J Pers Med. 2022 Sep 30;12(10):1615. doi: 10.3390/jpm12101615 (PMC9604616; doi:10.3390/jpm12101615)
Supplement: Supplementary file 1 [file jpm-12-01615-s001.zip › jpm-1891078-supplementary.pdf]

Supplemental Information

Supplement I. Survey questions regarding the acceptability of receiving pharmacogenomic (PGx) tests, attitudes toward data privacy and research, and acceptability of a statewide PGx database for clinical and research purposes.

1. Please rate the following statement: I would feel comfortable getting a pharmacogenomic test if recommended by a healthcare provider.
   Reminder: Pharmacogenomics is the study of how genes affect the way medications work in the body. It does not provide information regarding your ancestry or disease status.
   a. Strongly agree
   b. Agree
   c. Do not agree or disagree
   d. Disagree
   e. Strongly disagree

2. Please rate the following statement: I would be comfortable getting a pharmacogenomic test from a direct-to-consumer company or at a pharmacy that was not ordered by my physician or other healthcare providers.
   Reminder: Direct-to-consumer companies are commercial institutions not associated with your healthcare provider.
   a. Strongly agree
   b. Agree
   c. Do not agree or disagree
   d. Disagree
   e. Strongly disagree

3. If the option were available to me, the amount I would pay out-of-pocket for pharmacogenomic test would be:
   a. I would not pay any out-of-pocket money
   b. $0-$49
   c. $50-$149
   d. $150-$249
   e. $250-$499
   f. $500-$999
   g. $1000+

4. Please rate the following statement: I worry about the privacy of my pharmacogenomic data.
   a. Strongly agree
   b. Agree
   c. Do not agree or disagree
   d. Disagree
   e. Strongly disagree

5. Please rate the following statement: I would trust my healthcare professionals to keep my pharmacogenomic data private.
   a. Strongly agree
   b. Agree
   c. Do not agree or disagree
   d. Disagree

    e. Strongly disagree

6. Please rate the following statement: I would trust medical researchers to keep my pharmacogenomic data private.
    a. Strongly agree
    b. Agree
    c. Do not agree or disagree
    d. Disagree
    e. Strongly disagree

7. Please rate the following statement: I would trust genetic companies to keep my pharmacogenomic data private.
    a. Strongly agree
    b. Agree
    c. Do not agree or disagree
    d. Disagree
    e. Strongly disagree

8. Please rate the following statement: I believe we all can benefit if we share our pharmacogenomic data for research.
    a. Strongly agree
    b. Agree
    c. Do not agree or disagree
    d. Disagree
    e. Strongly disagree

9. Please rate the following statement: I would value a safe and private way to share my own information with researchers so they can study how pharmacogenomics influences the safety and effectiveness of medications.
    a. Strongly agree
    b. Agree
    c. Do not agree or disagree
    d. Disagree
    e. Strongly disagree

10. Do you agree with the following statement: I would value a statewide pharmacogenomic database for clinical use. This database would allow pharmacogenomic test results to be available to doctors and pharmacists, with an individual's permission, so they can check genes against medications prescribed to their patients for safety and effectiveness.
    a. Yes
    b. No
    c. I don't know

11. Do you agree with the following statement: I would value the opportunity to participate in a statewide pharmacogenomic database for clinical use, if I got the chance. "Clinical use" means my information could be provided to doctors and pharmacists, with my permission, so they could check my genes against drugs prescribed to me for safety and effectiveness.
    a. Yes
    b. No

c. I don’t know

12. Do you agree with the following statement: I would value a statewide pharmacogenomic database for research use. “Research use” means that individuals could allow their information to be provided to researchers, so they can study how pharmacogenomics influences safety and effectiveness of medications.
    a. Yes
    b. No
    c. I don’t know

13. Do you agree with the following statement: I would be likely to participate in a statewide pharmacogenomic database for research use, if I got the chance. This would allow my information to be provided to researchers, with my permission, so they can study how pharmacogenomics influences the safety and effectiveness of medications.
    a. Yes
    b. No
    c. I don’t know

Supplemental Figures

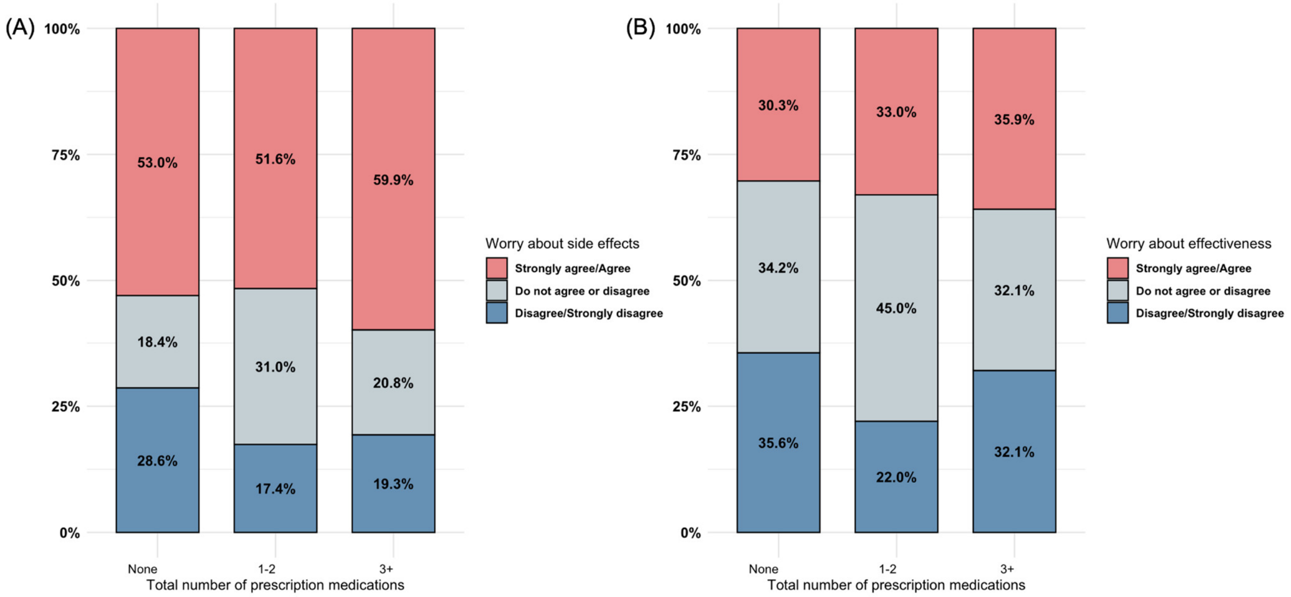

Figure S1. Safety and effectiveness concerns about medication use by the total number of prescription medications.

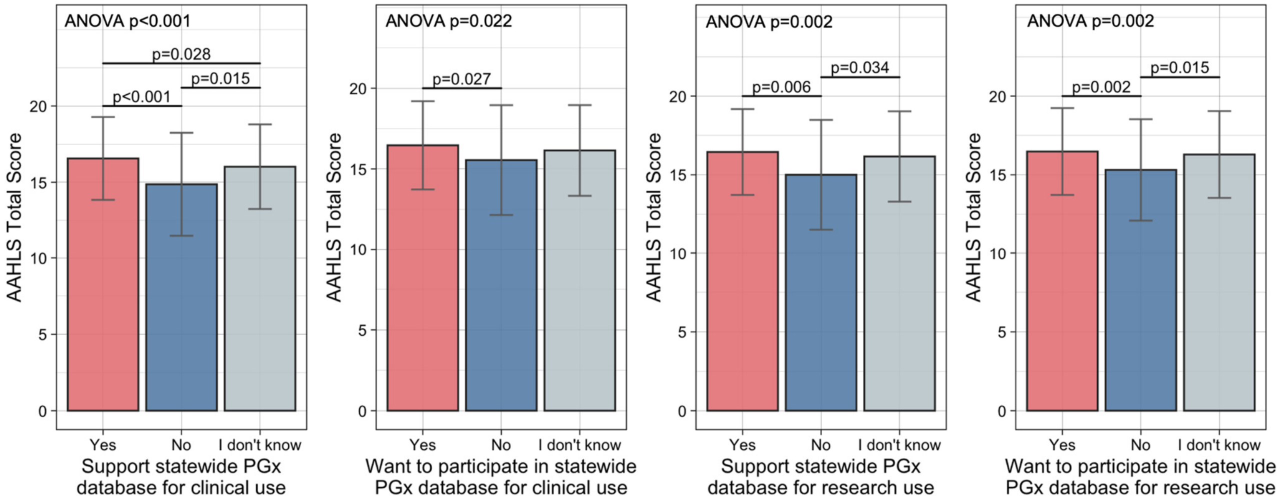

Figure S2. The associations between health literacy, assessed by AAHLS total score, and acceptability of a statewide pharmacogenomic database for clinical and research purposes among survey respondents (n = 763).
